# Evolution of the GII.3[P12] Norovirus from 2010 to 2019 in Jiangsu, China

**DOI:** 10.1186/s13099-021-00430-8

**Published:** 2021-05-26

**Authors:** Jianguang Fu, Jing Ai, Changjun Bao, Jun Zhang, Qingbin Wu, Liguo Zhu, Jianli Hu, Zheng Xing

**Affiliations:** 1 Medical School and the Jiangsu Provincial Key Laboratory of Medicine, Nanjing University, 22 Hankou Road, Gulou District, Nanjing, 210093 China; 2 Key Laboratory of Enteric Pathogenic Microbiology, Ministry of Health, Jiangsu Provincial Center for Disease Control and Prevention, Nanjing, China; 3 Suzhou Center for Disease Control and Prevention, Suzhou, China; 4 Soochow University Affiliated Children’s Hospital, Suzhou, China; 5 College of Veterinary Medicine, Department of Veterinary Biomedical Sciences, University of Minnesota At Twin Cities, Saint Paul, MN 55108 USA

**Keywords:** Norovirus, GII.3[P12], Evolution, Recombination, Mutations

## Abstract

**Objectives:**

Norovirus genotype GII.3[P12] strains have been an important pathogen for sporadic gastroenteritis infection. In previous studies of GII.3[P12], the number of specimens and time span are relatively small, which is difficult to truly reflect the infection and evolution of this type of norovirus. Here we report a molecular epidemiological study of the NoVs prevalent in Jiangsu between 2010 and 2019 to investigate the evolution of the GII.3[P12] strains in China.

**Methods:**

In this study 60 GII.3[P12] norovirus strains were sequenced and analyzed for evolution, recombination, and selection pressure using bioanalysis software.

**Results:**

The GII.3[P12] strains were continuously detected during the study period, which showed a high constituent ratio in males, in winter and among children aged 0–11 months, respectively. A time-scaled evolutionary tree showed that both GII.P12 RdRp and GII.3 VP1 sequences were grouped into three major clusters (Cluster I–III). Most GII.3[P12] strains were mainly located in sub-cluster (SC) II of Cluster III. A SimPlot analysis identified GII.3[P12] strain to be as an ORF1-intragenic recombinant of GII.4[P12] and GII.3[P21]. The RdRp genes of the GII.3[P12] showed a higher mean substitution rate than those of all GII.P12, while the VP1 genes of the GII.3[P12] showed a lower mean substitution rate than those of all GII.3. Alignment of the GII.3 capsid sequences revealed that three HBGA binding sites of all known GII.3 strains remained conserved, while several amino acid mutations in the predicted antibody binding sites were detected. The mutation at 385 was within predicted antibody binding regions, close to host attachment factor binding sites. Positive and negative selection sites were estimated. Two common positively selected sites (sites 385 and 406) were located on the surface of the protruding domain. Moreover, an amino acid substitution (aa204) was estimated to be near the active site of the RdRp protein.

**Conclusions:**

We conducted a comprehensive analysis on the epidemic and evolution of GII.3[P12] noroviruses and the results suggested that evolution was possibly driven by intergenic recombination and mutations in some key amino acid sites.

**Supplementary Information:**

The online version contains supplementary material available at 10.1186/s13099-021-00430-8.

## Background

Human norovirus has been recognized as the major cause for non-bacterial epidemic gastroenteritis [1, 2]. Norovirus is a non-enveloped RNA virus that contains a single-stranded genome segment of 7.5 kb encoding three open reading frames (ORFs). ORF1 encodes six non-structural proteins, including the RNA-dependent RNA polymerase (RdRp), whereas ORF2 and ORF3 encode structural proteins VP1 and VP2, respectively. VP1 is composed of shell (S) and protruding (P) domains with the latter being further divided into P1 and P2 domains [3]. Residues in the P2 domain are involved in the interaction with antibodies and histo-blood group antigens (HBGAs), which are supposed to be receptors or co-receptors of norovirus [4].

Up to now, noroviruses are phylogenetically classified into 10 genogroups (GI-GX) and, among them, at least five genogroups (GI, GII, GIV, GVIII and GIX) could infect humans [5]. GII.3 is a common cause for sporadic infection in infants and children as the second major genotype after GII.4 in China [6, 7]. To date, more reports focus on norovirus genotypes, such as GII.2[P16], GII.4, and GII.17[P17], that cause outbreaks. However, studies on the evolution of GII.3[P12] in detail are scarce [8–11]. In these studies, the number of specimens and time span are relatively small, making it difficult to truly reflect the infection and evolution of this type of norovirus. In this report, we performed a molecular evolutionary study on the GII.P12 RdRp and GII.3 VP1 regions of the noroviruses from fecal samples collected from pediatric patients from 2010 to 2019.

## Methods

### Stool specimens

Stool specimens were collected from hospitalized children with acute gastroenteritis in Suzhou Children’s Hospital during the period between January 2010 and December 2019 in Jiangsu, China. Acute gastroenteritis was defined to have at least three diarrheic stools and/or vomiting per day, caused by bacteria, viruses, fungi or parasites, but did not include cholera, dysentery, typhoid, or paratyphoid. The age of the participants was no old than 59 months, and the number of monthly samples was not less than 25. The specimen would be tested for norovirus, rotavirus, astrovirus and sapovirus but not for bacteria. All stool specimens were stored at −80 °C until use.

### Amplification of the RdRp and VP1 genes

Stool suspensions were prepared as 10% (w/v) in saline solution. Viral nucleic acid was extracted and tested for infection of NoVs by using MagMAXTM-96 Viral RNA Isolation Kit (Applied Biosystems, Foster City, CA) and the Qiagen Probe RT-PCR Kit (Qiagen, Hilden, Germany) on a 7500 real-time PCR platform (Applied Biosystems, Foster City, CA) with primers as described previously [12]. Norovirus-positive samples were detected with a region of 1095 bp targeted in the ORF1/ORF2 junction of the viral genome by one-step reverse transcription polymerase chain reaction (RT-PCR) for norovirus genotyping [12]. The norovirus genotyping tools used for analysis included the following resources online: http://www.rivm.nl/mpf/norovirus/typingtool and https://norovirus.ng.philab.cdc.gov.

Sixty GII.3[P12] strains from various times were selected for analyses. The complete RdRp (1.5 kb) and VP1 (1.7 kb) genomic fragments of the strains were amplified with GII.3[P12]-specific primers RdRp-12-F (forward: 5′-GGH GGY GAC RRC AAG GGV ACY TA-3′) and RdRp-12-R (reverse: 5′-TTC GAC GCC ATC TTC ATT CAC-3′), C-GII3-F (forward: 5′-CGA TCG CAA TCT GGC TCC CAG YT-3′) and C-GII3-R (reverse: 5′-AAT CCT GCT ATA AAA GCY CCA GCC-3′), respectively. All PCR products were purified and subsequently subjected to Sanger sequencing at the Sangon Biotech (Shanghai, China). Nucleotide sequences from the 60 GII.3[P12] strains sequenced for this study were deposited in GenBank under the accession numbers from MT678693 to MT678752.

### Evolutionary analyses

Nucleotide and amino acid (aa) sequence alignment was performed using BioEdit software. Nucleotide substitution models were identified using the Akaike information corrected criterion (AICc) implemented in JModelTest v2.1.7 [13]. The best of three clock models (strict clock, relaxed clock exponential and relaxed clock log normal) and three tree prior models (coalescent constant population, coalescent bayesian skyline and coalescent exponential population) were estimated using the path-sampling/stepping stone-sampling marginal likelihood estimation method. The optimal dataset was estimated as the relaxed clock exponential and exponential tree prior models for RdRp, relaxed clock log normal and bayesian skyline tree prior models for VP1, respectively (Additional file 1: Table S1). Convergence was evaluated by the effective sample size by Tracer v1.7, and values more than 200 were acceptable. Evolutionary analyses, including substitution rate, the time to most recent common ancestor (tMRCA), Bayesian skyline plot analysis (BSP) and phylogenetic analyses, were estimated using the relaxed clock exponential and the uncorrelated lognormal relaxed molecular clock, HKY (RdRp) and GTR + G (VP1) substitution, and Bayesian skyline coalescent models in BEAST v1.8.0 [14]. Markov chain Monte Carlo (MCMC) sample chains were run for 1.5 × 10^8^ steps for the RdRp and VP1 genes. The convergence of parameters was evaluated by Tracer v1.7.1, and FigTree v1.4.3 was used to view phylogenetic trees. The reliability of branches was supported by 95% HPDs. The relative frequency of amino acid occurrence (bits), grouped by amino acid chemistry (polar, neutral, basic, acidic, and hydrophobic) at informative sites were visualized using sequence logos (WebLogo v3; http://weblogo.threeplusone.com/) for each protein.

### Recombination detection analyses

Recombination detection was performed with the software package RDP v4.97 and Simplot 3.5.1 [15, 16]. RDP analysis was set up with an automated option including the following seven methods: RDP, GENECONV, BootScan, MaxChi, Chimaera, SiScan and 3Seq. For this study, only those sequences with events that were statistically significant (p ≤ 0.05) by at least five methods were reported as recombinants. The SimPlot analysis was performed by setting the window width and the step size to 200 bp and 20 bp, respectively.

### Selective pressure analysis

Datamonkey was used to determine specific positive and negative selected sites by rates of nonsynonymous and synonymous change (dN/dS) at every codon within the GII.3 VP1 protein [17]. The data set contained 163 aligned norovirus GII.3 VP1 sequences with 548 codons. Four methods, including single likelihood ancestor counting (SLAC), fixed effects likelihood (FEL), Fast, Unconstrained Bayesian AppRoximation (FUBAR), and mixed effects model of evolution (MEME), were chosen to detect selected sites. Sites under positive selection (dN > dS) were determined by a p value of  < 0.05 (SLAC, FEL, and MEME) and posterior probability of  > 0.9 (FUBAR). Negative selection sites (dN < dS) were estimated using SLAC, FEL, and FUBAR methods.

### Mapping of amino acid substitutions of the RdRp and P protein

A structural model of the capsid protein sequence in GII.P12 and GII.3 genotype was viewed and edited using PyMol v1.8. The RdRp and P protein crystal structures [Protein Data Bank (PDB) accession number 1SH0 and 6IR5] of the GII.P4 and GII.3 noroviruses (GenBank code: AJ583672 and U02030) were used to construct the dimer of the RdRp and P domain (including the P1 and P2 domains), respectively. Amino acid substitutions, conserved motif, active sites and RNA binding sites were mapped on the RdRp structure [18]. Positive selection sites and HBGA binding sites were mapped on the P domain structure.

### Statistical analyses

Data were type-entered into a database and analyzed using SPSS software version 18 (SPSS, Chicago, IL). Categorical variables were compared by Pearson χ^2^ test, Fisher’s Exact Test or Linear-by-Linear. Comparison between the two groups pairwisely was calculated by Bonferroni correction method. p < 0.05 were considered statistically significant.

## Results

### Temporal changes in the detection frequencies of the GII.3[P12] NoV

**Fig. 1:**
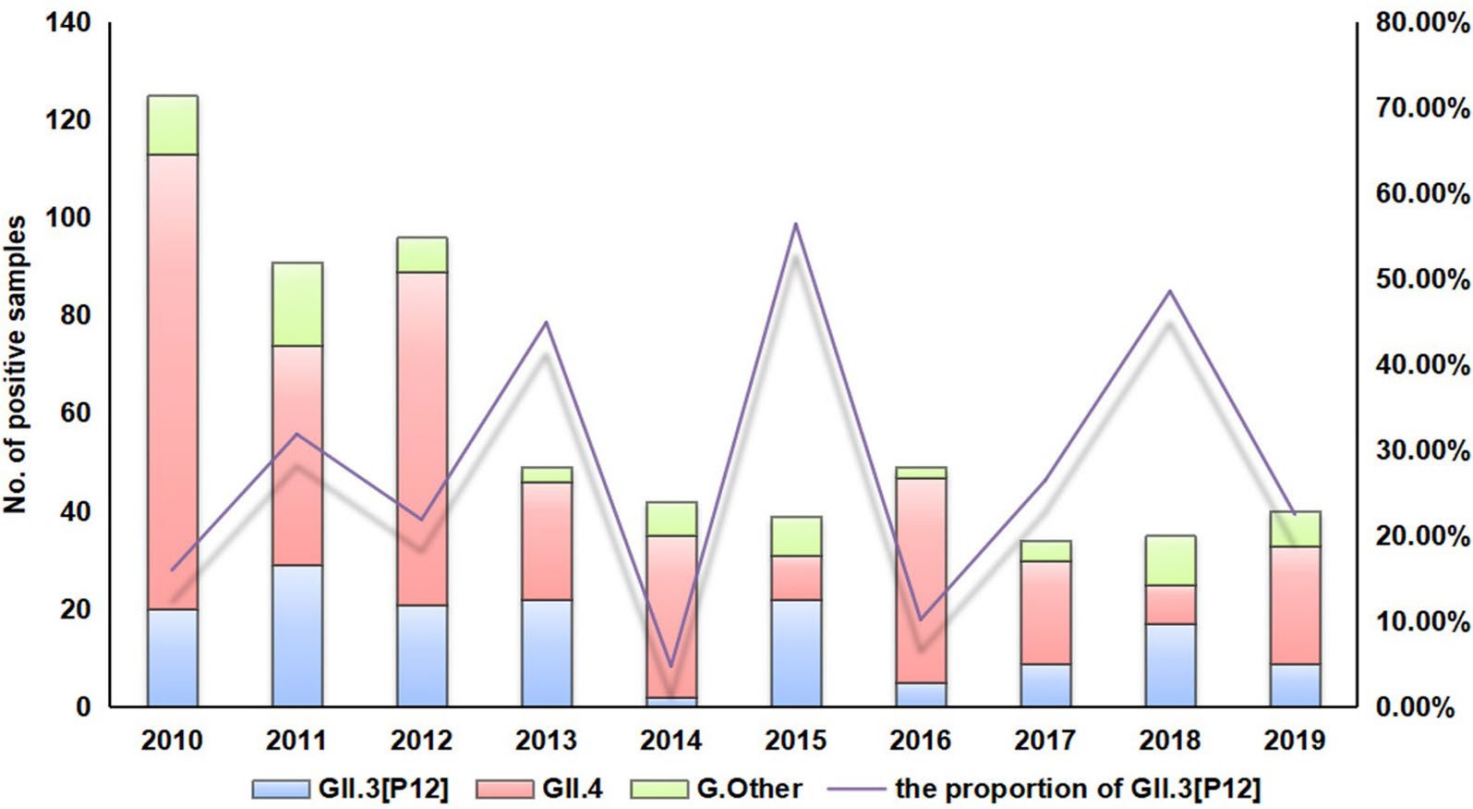
Distribution of the norovirus GII.3[P12] genotypes identified in sporadic cases of acute gastroenteritis in Jiangsu, China from 2010 to 2019. Bars represent the number of positive samples, and lines represent the proportion of GII.3[P12] genotypes in the positive samples

During the study period, a total of 3667 specimens submitted for the detection of NoV and other diarrhea viruses were included for analysis. Annual specimens ranged from 300 in 2017 to 431 in 2012. We analyzed the temporal changes of the genotypes of the NoV strains circulated from 2010 to 2019 (Fig. 1). The genotypes of the capsid and polymerase were determined in 600 strains, with 367 strains in GII.4 genotype (61.2%), 156 strains in GII.3[P12] genotype (26%), and 77 strains in other genotypes (12.8%). The GII.4 strains, including GII.4 Den Haag and GII.4 Sydney, had always been the dominantly epidemic strains except for the year of 2015 and 2018. The GII.3[P12] strains were continuously detected during the study period. High detection rates for the GII.3[P12] strains were observed in 2013, 2015 and 2018, but not in 2014 and 2016. Interestingly, in these 2 years GII.17[P17] and GII.2[P16] emerged as the non-GII.4 epidemic variants in Jiangsu, China.

### Epidemiological and clinical features

All of the samples were tested for norovirus, rotavirus, astrovirus and sapovirus but not for bacteria. Of the 600 genotyped NoV-positive samples, 380 were isolated from males and 220 from females, resulting in a male-to-female ratio of approximately 1.73:1 and considerably higher (2.25:1) with respect to GII.3[P12] (Table 1). GII.4 and other genotypes showed high constituent ratios among children aged 0–11 months and 12–23 months. However, GII.3[P12] showed a higher constituent ratio in age group 0–11 months (69.9%) than in 12–23 months (18.6%). NoVs could be detected throughout the year. The detection rate of GII.3[P12] was the highest in winter (43.8%, 42/96), followed by spring (34.2%, 26/76), and there was significant difference between the two seasons. GII.4 was the highest in autumn (71.1%, 185/258), followed by summer (65.3%, 111/170), and there was significant difference between the seasons as well. There was, however, no significant difference between seasons among the GII.Other strains. The clinical features of vomiting and fever were analyzed in diarrhea patients. In each genotype, the constituent ratio of these two clinical symptoms was similar, about 47% and 24% for vomiting and fever, respectively.

**Table 1:** Epidemiological and clinical features of GII.3[P12] norovirus

	GII.3[P12] (%) n = 156	GII.4 (%) n = 367	GII.Other (%) n = 77	Total (%) n = 600	Statistic	*p *value
Sex					4.967	0.083
Male	108 (69.2)	230 (62.7)	42 (54.5)	380 (63.3)		
Female	48 (30.8)	137 (37.3)	35 (45.5)	220 (36.7)		
Age*					41.583	0.000
0–11	109 (69.9)	172 (46.9)	29 (37.7)	310 (51.7)		
12–23	29 (18.6)	159 (43.3)	33 (42.9)	221 (36.8)		
24–35	10 (6.4)	22 (6.0)	6 (7.8)	38 (6.3)		
36–59	8 (5.1)	14 (3.8)	9 (11.6)	31 (5.2)		
Season*					47.555	0.000
Spring	26 (16.7)	37 (10.1)	13 (16.9)	76 (12.7)		
Summer	44 (28.2)	111 (30.2)	15 (19.5)	170 (28.3)		
Autumn	44 (28.2)	185 (50.4)	29 (37.6)	258 (43)		
Winter	42 (26.9)	34 (9.3)	20 (26.0)	96 (16)		
Symptom					0.499	0.779
Vomiting	73 (46.8)	173 (47.1)	38 (49.4)	284 (47.3)		
Fever	38 (24.4)	82 (22.3)	22 (28.6)	142 (23.7)		

The statistic value with * was calculated by using Bonferroni correction method in the comparison between the 0–11 months and 12–23 months group. The other statistic values were calculated by using Fisher’s Exact Test

According to the characteristics of the climate in Jiangsu, China, the seasons are divided as follows: spring (March–May), summer (June–August), autumn (September–November) and winter (December–February)

### Evolutionary analysis of the GII.P12 RdRp and GII.3 VP1 genes

**Fig. 2:**
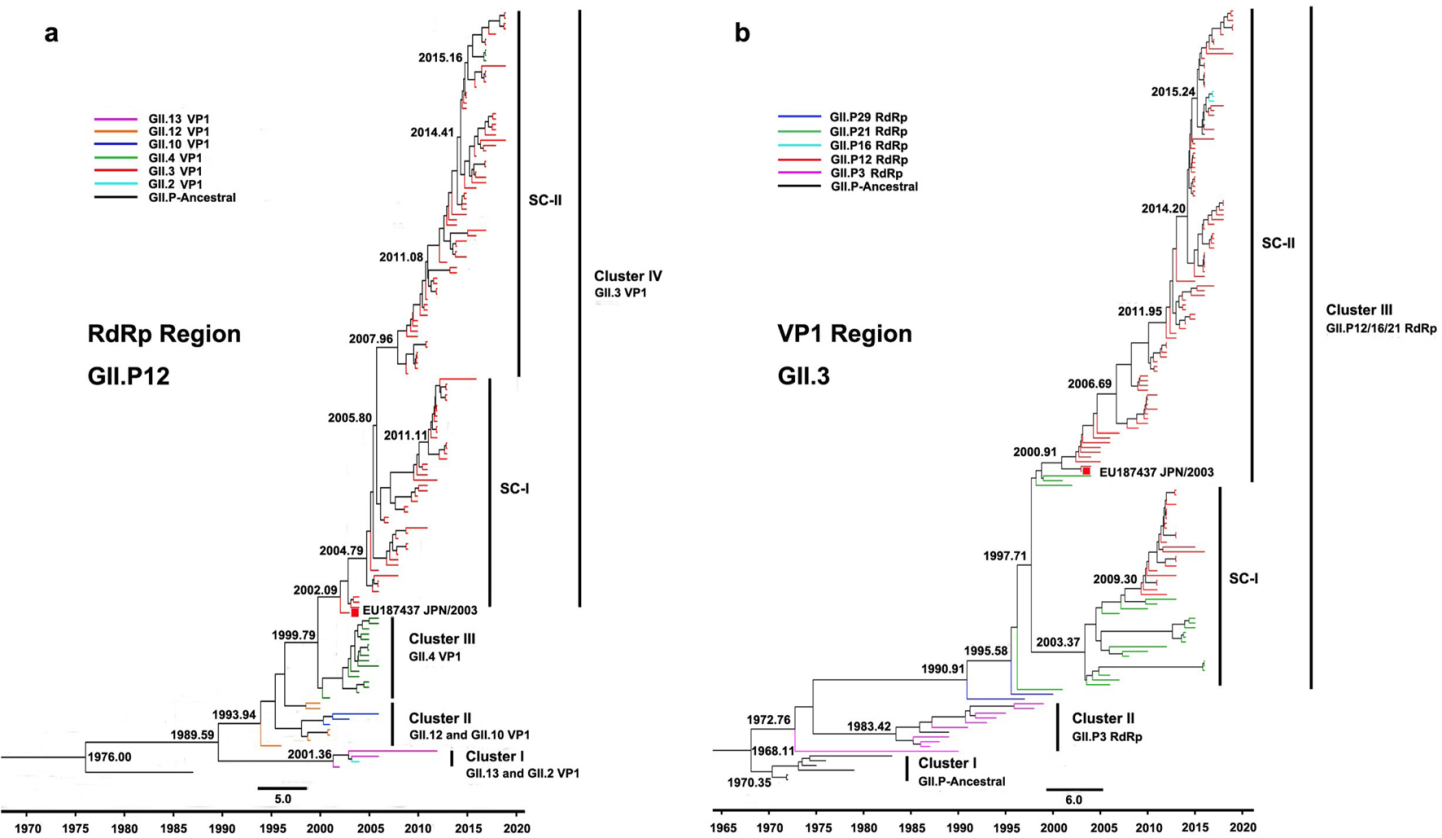
Markov chain Monte Carlo Bayesian phylogenetic trees of GII.P12 and GII.3 strains. **a** Trees were reconstructed using 144 partial RdRp nucleotide sequences of GII.P12 strains (780nt). **b** Trees were reconstructed using 163 full VP1 nucleotide sequences of GII.3 strains. The GII.P12 and GII.3 strains in the RdRp and VP1 trees are colored according to their capsid and polymerase genotype, respectively. Phylogenetic clustering is indicated by colors and names. Scale bar is actual time (years). The numbers after the decimal point at the node mean indicated average number of days per month, which is multiplied by 365 and further divided by 30 to determine the month

We constructed a time-scale evolutionary tree with the partial GII.P12 RdRp nucleotide (nt) sequences, including 84 RdRp sequences obtained from GenBank and 60 GII.3[P12] sequences collected in this study (Fig. 2a). Based on the relaxed clock exponential-RdRp sequences could be grouped into four major clusters (I, II, III and IV). Although all strains in this analysis had a GII.P12 RdRp gene except for an undefined ancestral RdRp genotype, six different VP1 genotypes were identified including GII.2, GII.3, GII.4, GII.10, GII.12 and GII.13. Cluster I and II included two VP1 genotypes, GII.13 and GII.2, GII.12 and GII.10, respectively. Cluster III contained the GII.4 VP1 genotype strains detected in 2001–2006. The GII.4 VP1 genotype strains had been rarely detected recently. However, three strains were isolated in Japan in 2017 (accession numbers: LC390332, LC421227 and LC421228.), demonstrating that the GII.4 VP1 genotype strain was still circulating. All GII.3 VP1 genotype strains fell in Cluster IV, which were split into two subclades (sub I and sub II). Cluster IV contained the strains mainly isolated in Japan, China, Russia and South Korea during the years from 2003 to 2019, suggesting that the GII.3[P12] appeared to be restricted to a specific geographic region, Asia. The earliest strain of GII.3 VP1 genotype strain deposited at GenBank was the one (EU187437) isolated in Japan in 2003. GII.3 VP1 genotype had become predominant in circulation since 2006. Overall, of the 84 strains from GenBank that were typed based on the VP1 gene sequence, the majority (51, 60.72%) possessed a GII.3 VP1, which were followed by the VP1 from GII.4 (19, 22.62%), GII.12 (6, 7.14%) and others (8, 9.52%).

We also used 60 full VP1 gene sequences of the GII.3[P12] noroviruses collected in our study and 103 GII.3 VP1 sequences available in the GenBank to generate a time-scale evolutionary tree. As Fig. 2b shows, the GII.3 VP1 gene phylogeny contained three clusters (Cluster I–III). Although all strains in this analysis had a GII.3 VP1 gene, six different RdRp genotypes were identified, which included GII.P3, GII.P12, GII.P16, GII.P21, GII.P29, and an undefined ancestral RdRp genotype. Strains in Cluster I included the undefined ancestral RdRp genotype and Cluster II had the genotype GII.3 RdRp. Strains collected from 1975 to 1983 were defined as ancestral. The accession number of them was KY442319, KY442320, HM072042, HM072045, HM072046 and PQ379713. GII.P29 genotype strains were not clustered because only a few of them were available with a complete VP1 gene, and strains, however, were closer to the ones in Cluster III in the evolutionary tree. Cluster III contained three RdRp genotypes (GII.P12, GII.P16, and GII.P21.) detected during the years from 2000 to 2019, which were further split into two subclades (Sub C I and II). Strains in Sub C I and II were associated with the GII.P12 and GII.P21 RdRp. Specifically, the GII.P12 genotype strains were mainly in Sub C II and the GII.P21 genotype strains in Sub C I. The earliest strain of the GII.3[P12] strain (EU187437) was also in Sub C II. The GII.P16 genotype strains belonged to Sub C II and have been recently detected in different VP1 genotypes, including GII.2, GII.4 and GII.13. Overall, of the 103 strains from GenBank typed based on the RdRp gene sequence, the majority (58, 56.31%) possessed a GII.P12 RdRp, which were followed by the strains in GII.P21 (23, 22.33%), GII.P3 (11, 10.68%), and others (11, 10.68%) RdRp.

### Molecular phylogenetic characteristics of recombinant noroviruses

**Fig. 3:**
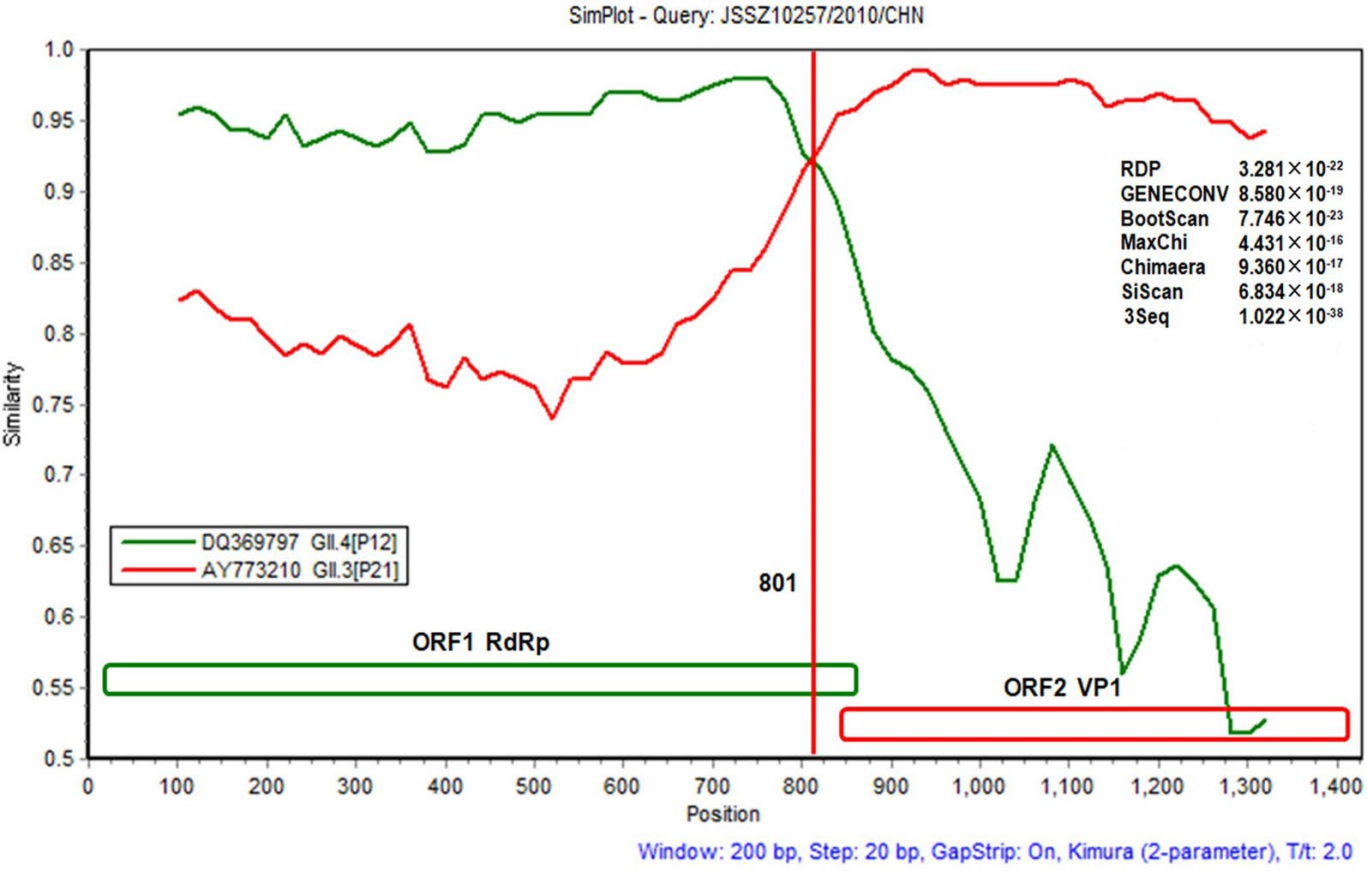
Recombinantion events identified by SimPlot analysis in this study. The breakpoint position of recombinant is marked with red line. The plot was obtained using a 200 bp sliding window, steps of 20 bp, and K-2-p model. The X-axis indicates the nucleotide positions in the multiple alignments of the NoV sequences; and the Y-axis indicates nucleotide identities (%) between the query sequence (JSSZ10257/2010/CHN) and the NoV reference strains. Seven methods (RDP, GENECONV, Chimaera, MaxChi, 3Seq, BootScan and SiScan) implemented in the RDP4 were also used to confirm the recombination events. The recombination event was confirmed as a significant (p < 0.01) with a Bootscan analysis in both the SimPlot and RDP4 programs

To characterize potential recombination events of the GII.3[P12] strains, a region of 1428 bp in the ORF1/ORF2 junction of the viral genome was analyzed by SimPlot v3.5.1 (Fig. 3). The GII.3[P12] strain was identified as an ORF1-intragenic recombinant of GII.4[P12] and GII.3[P21]. The recombination breakpoint was identified at position nucleotide 801, corresponding to the nucleotide positioned at 5033 in the whole viral genome, which was localized in the ORF1 region for the strain. The same recombination event was also detected with the RDP v4.97 software using seven different methods (RDP, GENECONV, Chimaera, MaxChi, 3Seq, BootScan, and SiScan), which was confirmed to be statistically significant (p values < 0.01) with a Bootscan analysis.

### Relative effective population size over time

**Fig. 4:**
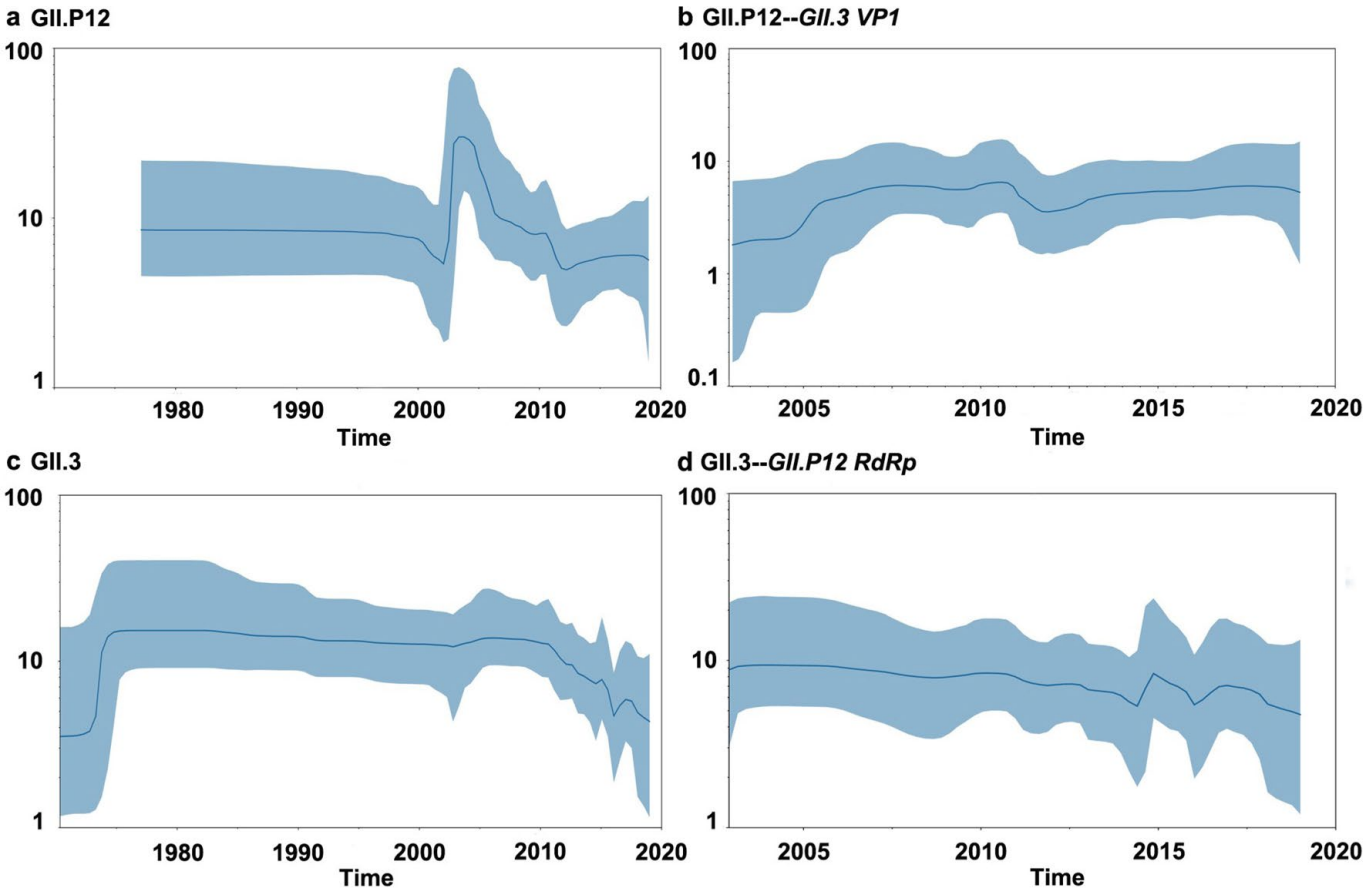
Bayesian skyline plot (BSP) for the RdRp and VP1 sequences of GII.3[P12]. Plots for all GII.P12 strains (**a**), GII.P12 with GII.3 VP1 (**b**), all GII.3 (**c**), GII.3 with GII.P12 RdRp (**d**) are shown. The y-axis represents the effective population size on a logarithmic scale, and the x-axis denotes the time in years. The solid blue line represents the median posterior value, and the blue area indicates the 95% highest probability density (HPD) intervals

A Bayesian skyline plot (BSP) coalescent model was used to measure the demographic history of the GII.P12 and GII.3 strains, which essentially plot Ne τ as a function of time. This model predicts the relative effective population size over time, when changes in effective population size reflect a change in genetic diversity. Notably, the effective population sizes of the GII.P12 strains (Fig. 4a) appeared to have two peaks, a large one around the year of 2003 and a small one around 2011, with two bottlenecks evident around 2002 and 2013. Considering the associated MCC tree (Fig. 2a), five different VP1 genotypes that comprised of the GII.2, GII.3, GII.4, GII.10 and GII.13 were observed in 2003, leading to a sudden gain of viral genetic diversity, which resulted in a steep increase in the skyline plot. The GII.P12 with the GII.3 VP1 genotype values (Fig. 4b) reached a peak around 2011, followed by a bottleneck around 2013, and then increased slightly, which coincided with the GII.P12 genotype as shown in Fig. 2a.

Population diversity was predicted to be better measured using the RdRp gene instead of the VP1 gene. The BSP obtained from the GII.3 capsid dataset (Fig. 4c) showed a pattern that was difficult to reconcile with the viral genetic diversity, which occurred to have five peaks (around 2005, 2010, 2012, 2015 and 2017) and two bottlenecks (around 2014 and 2016). The GII.3 with the GII.P12 RdRp genotype values (Fig. 4d) had five peaks and two bottlenecks, and the value decreased during the past decade, which coincided with the GII.3 genotype as shown in Fig. 2b.

### Gene evolution rates

The mean rate of nucleotide substitutions per site per year and the date of the most recent common ancestor were estimated separately for the GII.P12 RdRp and GII.3 VP1 data set based on the strict molecular clock model, which were listed in Table 2. The RdRp genes of the GII.3[P12] showed a mean substitution rate of 3.084 × 10^–3^ substitutions per site per year, which was higher than those of all GII.P12 (2.965 × 10^–3^ substitutions per site per year). On the other hand, the VP1 genes of the GII.3[P12] showed a lower mean substitution rate (3.866 × 10^–3^ substitutions per site per year) than those of all GII.3 (4.132 × 10^–3^ substitutions per site per year). The model estimated that the tMRCA of the GII.P12 RdRp and GII.3 VP1 data set existed around 1973, 2002, 1969 and 2001, respectively.

**Table 2:** Evolutionary parameters inferred by Bayesian analysis

Region	Genotype	Nucleotide substitution rate [× 10^–3^ (95%HPD)]	tMRCA [yr (95% HPD)]	Date of MRCA
RdRp	GII.P12	2.965 (2.302–3.696)	45.622 (36.815–56.687)	1973
	GII.3[P12]	3.084 (2.427–3.780)	17.155 (16.013–19.549)	2002
VP1	GII.3	4.132 (3.573–4.698)	49.892 (47.007–54.081)	1969
	GII.3[P12]	3.866 (3.119–4.618)	18.243 (16.046–21.290)	2001

### Amino acid alignment

**Fig. 5:**
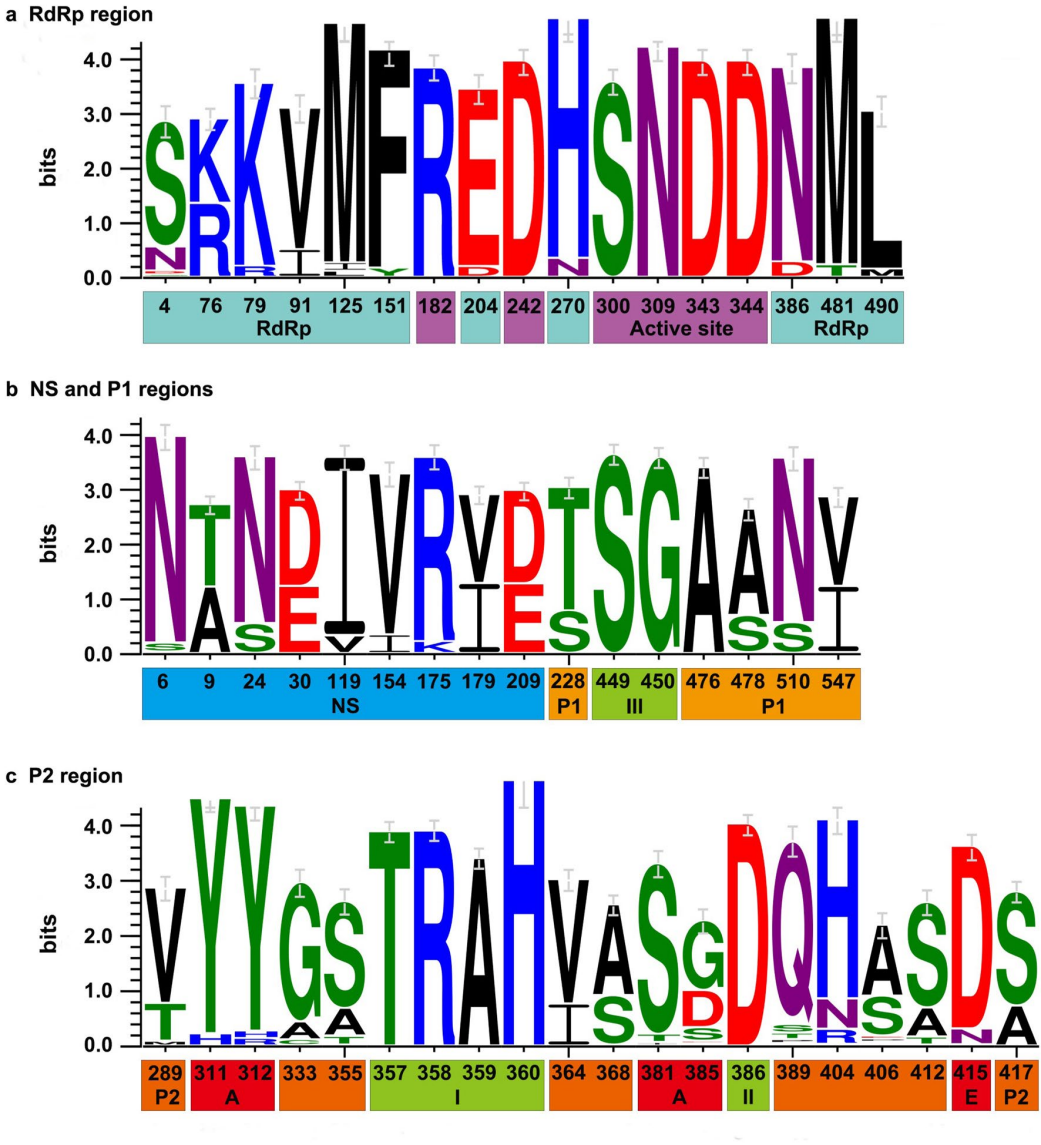
Bayesian informative amino acid sequences within the RdRp and VP1 region. Sequence logo of RdRp (**a**) amino acid sequences, NS, P1 (**b**) and P2 (**c**) of VP1 amino acid sequences derived from BLAST alignment (Fig. 2). The font size for each amino acid is proportional to percent conservation at each position. Bottom numbers indicate amino acid position and colors indicate the active sites (purple), predicted antibody binding sites (red) and HBGA binding sites (green). Sequence logos indicate the relative frequency of amino acid occurrence (bits) for locations where > 10% of sequences were identified with different strains. Amino acids have colors according to their chemical properties: polar amino acids (G, S, T, Y, C) show as green, neutral (Q, N) purple, basic (K, R, H) blue, acidic (D, E) red, and hydrophobic (A, V, L, I, P, W, F, M) amino acids as black

Informative amino acid sequences were aligned, including 122 complete RdRp sequences of the GII.P12 strains from 1996 to 2019 (Fig. 5a) and 163 VP1 sequences of the GII.3 strains from 1972 to 2019 (Fig. 5b, c). A conserved substitution was defined as an amino acid change (compared to a previous lineage) carried by the majority of strains within a cluster. Data showed that there were 11 unique amino acid substitutions in RdRp region, while conserved motif, active sites and RNA binding sites of all known GII.P12 strains remained conserved without change. A total of 27 unique amino acid substitutions were identified in VP1 protein, with 9 substitutions in NS domain, 5 in P1 domain, and 13 in P2 domain. While three HBGA binding sites of all known GII.3 strains remained conserved, several amino acid mutations were found in the predicted antibody binding sites as shown in Fig. 5. Sites 385, 389, and 406 may be important evolutionary sites since these sites were highly variable with three or four different conserved substitutions. In particular, site 385 was found peripheral to the HBGA-binding site II, also located in the predicted antibody binding sites, which may have an important effect on the viral antigenicity. Several unique amino acid mutations for SC-II-P12 of Cluster III (site 6, 175, 179 and 209) were found in NS region. Interestingly, more unique amino acid mutations for SC-I of Cluster III were found in NS region (site 24), P1 region (site 228, 476 and 510) and P2 region (site 364, 412 and 417), respectively.

### Estimation of positive and negative selection sites in VP1

Selection pressure on each site in the capsid gene was analyzed for the GII.3 and GII.3[P12] strains (Table 3). Sites under diversifying or purifying selective pressure were determined based on rates of synonymous and nonsynonymous change. Positively selected sites were estimated by four methods: single likelihood ancestor counting (SLAC), fixed effects likelihood (FEL), Fast and Unconstrained Bayesian AppRoximation (FUBAR), and mixed effects model of evolution (MEME). 13 and 6 sites under positive selection were detected, respectively. Most of the sites were located within the surface of the capsid protein. The results suggested that selective pressure from the host caused amino acid substitution of the virus. We also detected sites 214, 281, and 390 in GII.3 strains and sites 68, 135, and 247 in GII.3[p12] strains under negative selection by the SLAC, FEL, and FUBAR methods, respectively. Common sites under positive selection estimated by the four methods occurred after amino acid changes at site 385 in both of the strains. Except for the SLAC method for the GII.3[P12] strains, site 406 was also the common site under positive selection.

**Table 3:** Positive selection sites on capsid gene in human NoV GII.3. Cut off p value < 0.05

VP1	Method	Residue number														
		7	24	68	179	200	258	364	368	385	389	404	405	406	543	547
GII.3	SLAC									*	*			*		
	FEL								*	*	*			*		
	FUBAR									*	*	*		*		*
	MEME	*		*		*	*	*		*	*	*	*	*	*	*
GII.3[P12]	SLAC									*						
	FEL				*					*				*		
	FUBAR		*		*					*				*		*
	MEME						*			*				*		

### Mapping of amino acid substitutions of the RdRp and P protein

**Fig. 6:**
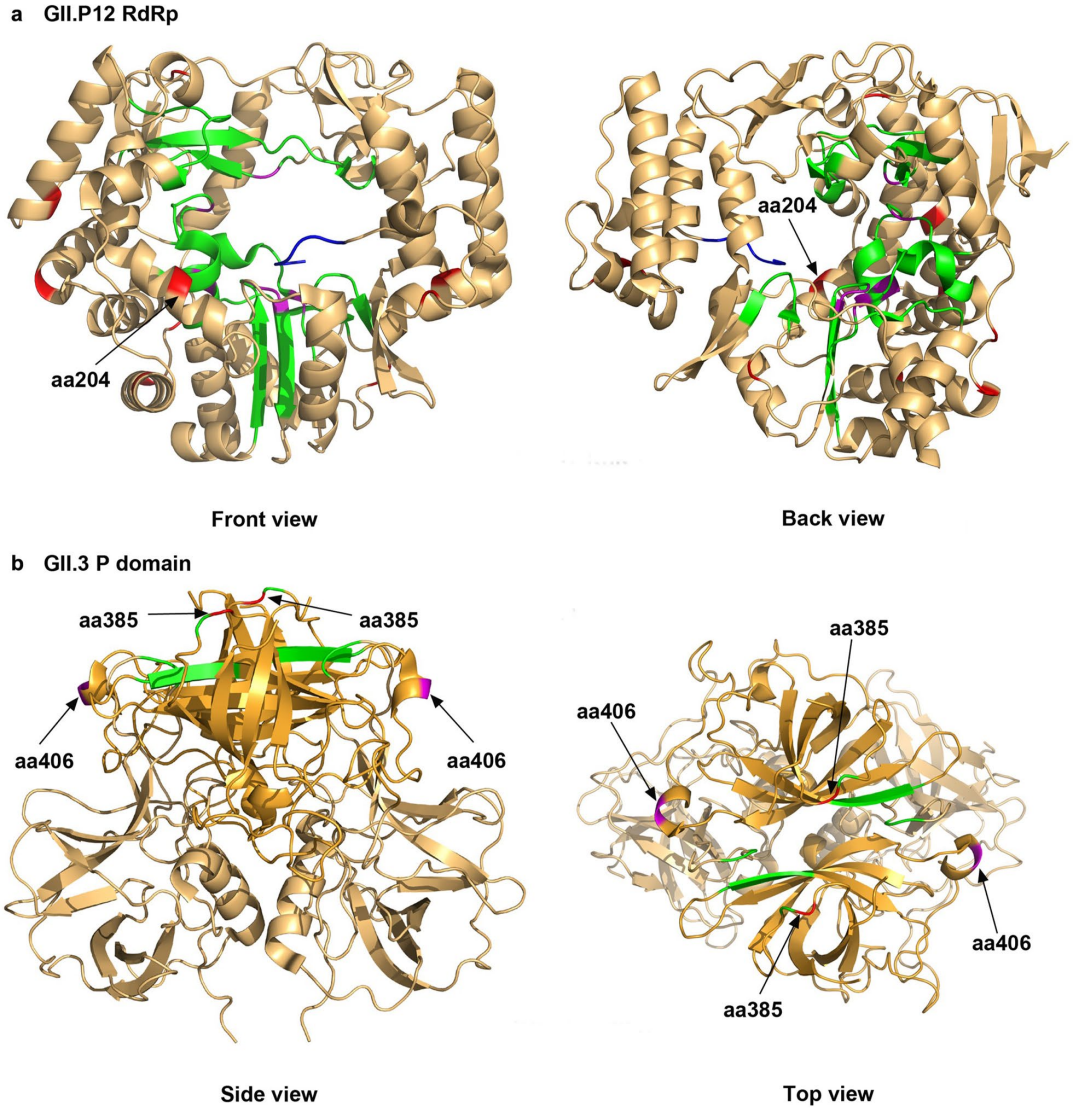
Structural models of the RdRp and P protein for representative GII.3[P12] strains. Homology structures of the RdRp of GII.P4 [PDBID 1SH0, **a**] and the P domain of GII.3 [PDBID 6IR5, **b**] are shown. The RdRp structure is shown from the front view and back view. Amino acid substitutions are colored red. The conserved motif, active sites and RNA binding sites were colored green, purple and blue, respectively. The P domain structure is shown from the top view and side view. Two positively selected sites are colored red and purple, respectively. The HBGA binding sites are colored green. The accession numbers for the sequences of the RdRp of GII.4 and the P domain of GII.3 were AJ583672 and U02030, respectively

In order to evaluate the possible effect of substitutions on protein function, we mapped the amino acid substitutions of GII.P12 RdRp genotypes onto three-dimensional structures of the RdRp protein (Fig. 6a). A total of 11 amino acid substitutions were identified among the GII.P12 strains. Of these, the substitution at aa204 was located close to the active site and no substitution was located close to RNA binding site (Fig. 6a). Furthermore, we mapped the HBGA binding sites in Fig. 5B, C (green color) and two common positively selected sites in Table 3 onto the GII.3 capsid dimer three-dimensional structure (Fig. 6b). The sites under positive selection were located on the surface of the protruding domain. Site 385, a predicted antibody binding site, was adjacent to site 386, an HBGA binding site. Of significance, the substitutions occurring at these sites may result in the emergence of the current epidemic GII.3 variant strains. These results suggest that selective pressure from hosts caused amino acid substitution of the virus.

## Discussion

During our study period, the GII.3[P12] strains had never caused a large-scale outbreak. They had been involved in sporadic epidemics all the time. Many strains had disappeared or been replaced by newer ones over time and even GII.4 strains were replaced by new subtypes. Although the detection rate of the GII.3[P12] strains was secondary to GII.4, they had never been replaced and disappeared. The GII.4 genotype had been dominant globally for two decades with the most recent one, Sydney2012, emerging in 2012 [19]. As the first non-GII.4 epidemic variant, GII.17 caused an increasing number of outbreaks in 2014/15 in China [11, 12]. In 2016/17, another non-GII.4 epidemic variant, the GII.2[P16] strain, emerged and rapidly became the predominant genotype throughout the mainland China [9, 10]. GII.3[P12] was detected with high detection rates in 2013, 2015 and 2018, and low detection rates in 2014 and 2016. It seemed that the detection rate of GII.3[P12] decreased at the beginning when new epidemic strains emerged and increased significantly 1–2 years later. With an exception, GII.4 Sydney[P31] did not lead to an obvious decrease of GII.3[P12] in 2012, probably because infection of the GII.4 and GII.3 genotypes might have co-existed in the population for a long time, reaching a balance to certain degree. The emergence of the new GII.4 subtypes somehow did not tip the balance. A recent report suggested that genotype specific herd immunity in infants and young children lasted for at least a few years, thereby influencing the endemic NoV genotype in the next season [20]. This observation is consistent with our results in this report. The detection rate of GII.3[P12] increased every other year, which rarely led to an explosive epidemic.

Based on RdRp and VP1 gene sequences, the branching pattern was shown to be associated with intergenic recombination within the ORF1/ORF2 region. Both GII.P12 and GII.3 strains had evolved under the influence of several recombination events. The earliest strain of the GII.3[P12] genotype was the strain EU187437 isolated in Japan in 2003, which could be derived from the recombination of GII.4 [P12] and GII.3 [P21] strains in 2000–2002. Additionally, phylogenetic analysis indicates that, after their initial emergences in 2003, the GII.3[P12] noroviruses underwent rapid genetic diversification. The GII.3 [P21] strains evolved into two subclades and the corresponding GII.3[P12] formed two subclades in the evolution. Previous studies also showed that GII.3 VP1 sequences could be grouped into three major clusters, in which clusters II and III were compatible with the present SC I and SC II [21]. As both SC I and SC II contained the strains isolated in late 2010, it was likely that acquisition of a different RdRp gene allowed simultaneous evolution along with two distinct subclades. The clear separation of SC2 from SC1 suggested that the GII.3[P12] noroviruses may be highly diverse. The GII.3[P12] strains in SC I were the main causes for epidemics throughout China and Korea from 2006 to 2013, while the SC2 viruses exhibited a broadest time span distribution from 2003 to 2019 and geographic distribution in China, Japan, Korea, France, Russia and the United States, suggesting that it had an advantage in global spread. The GII.4[P12] and GII.3[P16] genotype strains isolated in 2017 also belonged to SC II. In particular, the GII.P16 genotype strains, recently detected in different VP1 genotypes, included GII.2, GII.4 and GII.13 [22]. The GII.2[P16] and GII.4 Sydney[P16] strains were detected around the globe in 2015–2016 [23], which had been the predominant strains that caused local norovirus outbreaks. The recombination of GII.3 with the RdRp gene of the current popular strain may be a beginning of a new trend for NoV evolution. However, no more recombinant strains of this genotype have been found up to date.

The fitness of a virus is influenced by its genetic diversity [24]. GII.4 noroviruses have predominated for more than 20 years [1], which suggests that GII.4 noroviruses may possess greater viral fitness in human populations as compared to other norovirus genotypes [25]. GII.4 norovirus has a high VP1 gene evolutionary rate (5.33 × 10^–3^), which may increase its ability to evade immune responses [26]. The VP1 gene of some rare genotypes, such as GII.17 and GII.2, showed considerable genetic diversity and evolved at a high rate of substitutions/site/year due to its possible acquisition of a novel polymerase [11, 22]. We compared the substitution rate of GII.3[P12] associated with the GII.P12 RdRp and GII.3 VP1. Over time the GII.P12 and GII.3 genes had evolved under the influence of several recombination events, thus it was unlikely that the gene had evolved at a steady rate. As shown in Table 2, after the GII.3[P12] recombination, mean substitution rates of the GII.P12 RdRp increased while those of the GII.3 VP1 decreased. Because the 95% HPD scores were overlapping, p > 0.05, there was no statistical difference between the rates. However, we could still make some suppositions from the trend. The result may suggest that the recombination was more beneficial to the evolution of GII.P12 RdRp than to GII.3 VP1. The increased substitution rate conferred by the recombinant GII.3 VP1 probably produced a fitter virus, thus the GII.3[P12] emerged as a prevalent pediatric strain.

The substitution rate is a measurement of the underlying mutation rate combined with selective and transmission pressures but not a direct measurement of the mutation rate conferred by the RdRp [27]. An analysis of the substitution rate in the RdRp gene (rather than VP1) may better serve as an indication of the underlying RdRp mutation rate, since the RdRp protein should be less exposed to strong selective pressures [26]. Unlike GII.3 VP1, partial GII.P12 RdRp gene sequence was analyzed as a measure of the substitution rate, due to a low sample size with complete gene sequences. A previous report showed that densely sampled short RdRp sequences provided more reliable evolutionary data than fewer longer sequences [26]. In the present study, the VP1 genes of the GII.3 showed a lower mean substitution rate (4.132 × 10^–3^ substitutions per site per year) than in a previous report (4.82 × 10^–3^ substitutions per site per year) [21]. Several factors, such as sequence numbers, methodology, and collection dates, may affect the differences in the evolutionary rates between the previous and present data.

The effective population size may reflect virus genome populations in the host, which is predicted to be better measured using the RdRp gene instead of the VP1 gene [28]. Our results were in line with this view. Compared with GII.3 VP1 dataset, the BSP obtained from the GII.P12 RdRp dataset was easier to understand. The greatest peak in the population size was observed following a large bottleneck just prior to 2003, which was likely due to the gain of five different VP1 sequences. The population size of the polymerase type increased to the year when the VP1 genotypes of the strains were obtained, which was consistent with a previous report [25]. The second bottleneck around 2013, after a small peak around 2011, occurred probably because the number of the strains in both SCI and SCII increased significantly around 2011, while the strains in the SCI were rarely detected after 2013.

The driving force for the GII.3[P12] norovirus evolution was not only intergenic recombination but also amino acid substitutions [29]. A previous study showed that the amino acids around the active sites regulate viral genome replication [30]. GII.P12 contains 11 amino acid substitutions, one (aa204) of which is adjacent to the RdRp active sites. The amino acid substitutions in the capsid protein primarily occurred, which was responsible for changed antigenicity and infectivity of the strains [31]. Three of 27 substitutions in the VP1 protein, sites 385, 389, and 406, were more prominent which were highly variable with three or four different conserved substitutions. These three sites had also been identified under positive selection.

Host defense mechanisms may lead to virus immune escape with mutations which are thought to the result of the positive selection [28]. We used four methods (FEL, IFEL, SLAC, and FUBAR) to make a candidate list of positively selected amino acid sites. The SLAC method is appropriate for detecting non-neutral evolution and may be a stricter algorithmic model for estimating positive selection sites [28]. Positive selection was estimated to occur at fifteen sites with amino acid substitutions even though the SLAC method only identified three of them. These results indicate that they were likely to be major sites of selection at a population level. Based on these four methods, two sites at 385 and 406 under positive selection were estimated in all GII.3 strains and GII.3[P12] strains, positioned on the outer surface loops of the protruding P2 domain in the VP1. The site 389 was not considered as a positive selection site in the GII.3[P12] strains. As shown in Table 3, there were no amino acid substitutions at site 389 in the GII.3[P12] strains, which was in accordance with the result of the positive selection. Site 385 under the positive selection was peripheral to the HBGA-binding site II, which was also located in the predicted antibody binding sites. A single amino acid change may be sufficient to cause immune escape from immunity [32]. A high variability of this site may be due to the pressure under immune selection.

## Conclusion

In conclusion, we report an evolutionary analysis of the epidemic spread of the norovirus GII.3[P12] strains in samples collected from the epidemic spread over a period of 10 years in Jiangsu province. Multiple genetic signatures linked to substitution rate, selective pressure, HBGA binding, and antigenic epitopes were identified. Our data indicate that the evolution of the GII.3[P12] strains was driven by intergenic recombination and amino acid substitutions. Faster evolutionary rates of the GII.P12 than GII.3 VP1 after the GII.3[P12] recombination revealed that the recombination was more beneficial to the evolution of the GII.P12 RdRp than the GII.3 VP1. Considering norovirus GII.3[P12] is still rapidly evolving after current epidemic spread and has been sporadically detected outside Asia, close attention to the mechanisms driving the evolution of norovirus strains is important for future noroviral control and prevention.

## Supplementary Information

**Additional file 1: Table S1.** Clock and prior models test using pass sampling or stepping-stone sampling method.

## Data Availability

All data involved in this study is available upon reasonable request made to the corresponding author.
