# Supplementary material for: Evolution of the GII.3[P12] Norovirus from 2010 to 2019 in Jiangsu, China
Source: Gut Pathog. 2021 May 26;13:34. doi: 10.1186/s13099-021-00430-8 (PMC8149921; doi:10.1186/s13099-021-00430-8)
Supplement: Supplementary file 1 — Additional file 1: Table S1. Clock and prior models test using pass sampling or stepping-stone sampling method. [file 13099_2021_430_MOESM1_ESM.docx]

**Table S1. Clock and prior models test using pass sampling or stepping-stone sampling method.** The best clock model and prior model are shown in bold.

| **Molecular clock model** | **Coalescent tree prior** | **Log marginal likelihood** | |
| --- | --- | --- | --- |
| **GII.P12 RdRp** | | |  |
| Strict clock | Constant size | -4821.386 |  |
| Strict clock | Exponential growth | -4521.616 |  |
| Strict clock | Bayesian skyline | -4516.213 |  |
| Uncorrelated exponential relaxed clock | Constant size | -4494.129 |  |
| **Uncorrelated exponential relaxed clock** | **Exponential growth** | **-4490.988** |  |
| Uncorrelated exponential relaxed clock | Bayesian skyline | -4494.767 |  |
| Uncorrelated lognormal relaxed clock | Constant size | -4645.358 |  |
| Uncorrelated lognormal relaxed clock | Exponential growth | -4496.767 |  |
| Uncorrelated lognormal relaxed clock | Bayesian skyline | -4504.427 |  |
| **GII.3 VP1** | | |  |
| Strict clock | Constant size | -13521.345 |  |
| Strict clock | Exponential growth | -13539.613 |  |
| Strict clock | Bayesian skyline | -13527.500 |  |
| Uncorrelated exponential relaxed clock | Constant size | -13477.049 |  |
| Uncorrelated exponential relaxed clock | Exponential growth | -13488.695 |  |
| Uncorrelated exponential relaxed clock | Bayesian skyline | -13479.528 |  |
| Uncorrelated lognormal relaxed clock | Constant size | -13474.555 |  |
| Uncorrelated lognormal relaxed clock | Exponential growth | -13469.003 |  |
| **Uncorrelated lognormal relaxed clock** | **Bayesian skyline** | **-13465.138** |  |
